# Globoside accelerates the differentiation of dental epithelial cells into ameloblasts

**DOI:** 10.1038/ijos.2016.35

**Published:** 2016-10-21

**Authors:** Takashi Nakamura, Yuta Chiba, Masahiro Naruse, Kan Saito, Hidemitsu Harada, Satoshi Fukumoto

**Affiliations:** 1 Division of Molecular Pharmacology and Cell Biophysics, Department of Oral Biology, Tohoku University Graduate School of Dentistry, Sendai, Japan; 2 Division of Pediatric Dentistry, Department of Oral Health and Development Sciences, Tohoku University Graduate School of Dentistry, Sendai, Japan; 3 Division of Developmental Biology and Regenerative Medicine, Department of Anatomy, Iwate Medical University, Yahaba, Japan

**Keywords:** ameloblast, differentiation, enamel matrix, epiprofin, glycosphingolipids, tooth development

## Abstract

Tooth crown morphogenesis is tightly regulated by the proliferation and differentiation of dental epithelial cells. Globoside (Gb4), a globo-series glycosphingolipid, is highly expressed during embryogenesis as well as organogenesis, including tooth development. We previously reported that Gb4 is dominantly expressed in the neutral lipid fraction of dental epithelial cells. However, because its functional role in tooth development remains unknown, we investigated the involvement of Gb4 in dental epithelial cell differentiation. The expression of Gb4 was detected in ameloblasts of postnatal mouse molars and incisors. A cell culture analysis using HAT-7 cells, a rat-derived dental epithelial cell line, revealed that Gb4 did not promote dental epithelial cell proliferation. Interestingly, exogenous administration of Gb4 enhanced the gene expression of enamel extracellular matrix proteins such as ameloblastin, amelogenin, and enamelin in dental epithelial cells as well as in developing tooth germs. Gb4 also induced the expression of TrkB, one of the key receptors required for ameloblast induction in dental epithelial cells. In contrast, Gb4 downregulated the expression of p75, a receptor for neurotrophins (including neurotrophin-4) and a marker of undifferentiated dental epithelial cells. In addition, we found that exogenous administration of Gb4 to dental epithelial cells stimulated the extracellular signal-regulated kinase and p38 mitogen-activated protein kinase signalling pathways. Furthermore, Gb4 induced the expression of epiprofin and Runx2, the positive regulators for ameloblastin gene transcription. Thus, our results suggest that Gb4 contributes to promoting the differentiation of dental epithelial cells into ameloblasts.

## Introduction

During tooth development, interactions between dental epithelial and mesenchymal cells play important roles in cell proliferation and differentiation to regulate the size and shape of a tooth.^1^ A variety of growth factors and their receptors are involved in these interactions,^2^ and the morphological formation of the tooth crown is precisely regulated by a balance between the proliferation and differentiation of dental epithelial cells.^3^ Enamel, the hardest structure in the human body, covers the entire tooth surface to prevent attrition. Dental epithelial cell-derived ameloblasts are responsible for enamel formation by secreting enamel matrix molecules, including ameloblastin (Ambn), enamelin (Enam), and amelogenin (Amel), as well as enamel matrix-specific proteases, such as kallikrein 4 and matrix metalloproteinase-20 (Mmp-20).^4, 5, 6, 7, 8^ Disruption of the production of enamel matrix molecules or their proteases leads to enamel hypoplasia.^9^ For example, Ambn-null mice display severe enamel hypoplasia that is accompanied by detachment of the ameloblasts from the enamel matrix and a loss of their polarity.^10^ In addition, Ambn-null mice develop odontogenic tumours because of the re-entrance of premature ameloblasts into the cell cycle.^10^ Epiprofin (Epfn) is a master transcription factor of ameloblasts and is continuously expressed in dental epithelial cells. Epfn promotes Ambn gene expression by binding to its promoter region.^11, 12, 13, 14^ Thus, Epfn-null mice fail to develop enamel structures because of a blockage of dental epithelial cell differentiation into ameloblasts.^15, 16^ Epfn also regulates the cell cycle exit by interacting cell cycle regulators.^17^ The transcription factor Runx2 (also called Cbfa1), known for its critical role in osteoblast differentiation, has also been shown to regulate Ambn gene transcription by binding to the osteoblast-specific element in the proximal Ambn promoter region.^18^

Four neurotrophins in mammals, nerve growth factor (NGF), brain-derived neurotrophic factor, neutrophin (NT)-3, and NT-4/5, act *via* the receptors p75NTR, TrkA, TrkB, and TrkC, respectively.^19, 20^ During tooth development, NT-4 and its receptor, TrkB, play important roles in the late stage of tooth development, during which immature dental epithelial cells differentiate into enamel-forming ameloblasts. NT-4 also promotes the differentiation of dental epithelial cells into ameloblasts *via* TrkB-FL, a nerve growth factor receptor.^21^ Differentiating dental epithelial cells express unique glycosphingolipids (GSLs) such as GM3 in acidic fractions, as well as Gb4 and lactosylceramide (LacCer) in neutral fractions. GM3 and LacCer play important roles to induce nerve growth factor NT-4-mediated differentiation of dental epithelial cells into ameloblasts.^22^ However, the role of Gb4 during tooth development remains unclear.

**Figure 1:**
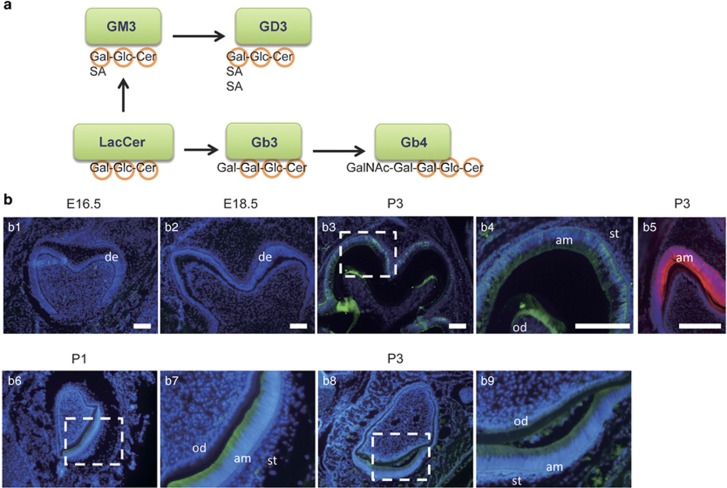
**The globoside synthesis and the expression of Gb4 in developing mouse molars.** (**a**) Glycosphingolipids are synthesized by the sequential action of glycotransferases that are initiated from the glycosylation of ceramide followed by the synthesis of lactosylceramide, a common precursor of most glycosphingolipids, including Gb4. By the successive addition of sialic acid residues onto *via* the sialyltransferases Sial-T1 and Sial-T2, the GM3 and GD3 gangliosides are synthesized, which are also important in ameloblast differentiation. (**b**) An immunofluorescence analysis showed the localization of Gb4 in the lower first molars obtained on (**b**1) embryonic day 16.5 (E16.5), (**b**2) E18.5, and (**b**3) postnatal day 3 (P3). (**b**4) An enlarged image of the dotted box in (**b**3). (**b**5) A positive control for AMBN staining in the lower first molar on P3. (**b**6, **b**7) Gb4 staining of incisors from P1 and P3 (**b**8, **b**9) mice with anti-Gb4 (green) antibodies. Nuclear staining was performed with DAPI dye (blue). Bar=100 μm. am, ameloblast; DAPI, 4′,6-diamidino-2-phenylindole; de, dental epithelium; Gb4, globoside; LacCer, lactosylceramide; od, odontoblast; qPCR, quantitative polymerase chain reaction; st, stratum intermedium.

GSLs are ubiquitously expressed in all eukaryotic cells and form clusters that mainly localize in the outer leaflet of the plasma membrane.^23^ Because clustered GSLs at the cell surface membrane interact with functional membrane proteins such as integrins, growth factor receptors, and tetraspanins, they are involved in a variety of cellular physiological processes, including cell adhesion, growth, motility, and cell-fate determination or differentiation.^24, 25, 26, 27^ Ganglioside biosynthesis begins with ceramide formation that takes place in the endoplasmic reticulum. This is followed by the synthesis of glucosylceramide (GlcCer). LacCer is synthesized by the GalT-1 enzyme from GlcCer, and GM3 is synthesized by α2,3-sialyltransferase (GM3S) from LacCer. On the other hand, globotriaosylceramide (Gb3) is synthesized by the Gb3/CD77 enzyme (a1, 4Gal-T) from LacCer, and Gb4 is synthesized by the enzyme (b1, 3 GalNac-T) from Gb3^28^ (Figure 1a).

In this study, we found that Gb4 is involved in the differentiation of dental epithelial cells by controlling the expression profiles of receptors for NT-4, one of the ameloblast inducers.

## Materials and methods

### Cell cultures and conditions

HAT-7, a rat-derived dental epithelial cell line, was maintained as described previously.^29^ Briefly, cells were cultured in Dulbecco's modified Eagle's medium (DMEM)/F-12 (Invitrogen, Carlsbad, CA, USA) supplemented with 10% fetal bovine serum (FBS), 100 U·mL^−1^ penicillin G, and 100 μg·mL^−1^ streptomycin (Invitrogen, Carlsbad, CA, USA) in a humidified atmosphere containing 5% CO_2_ at 37 °C. For the gene expression analyses, 2.0 × 10^5^ cells per well were seeded into 6-well plates and cultured with or without 0.5 μmol·L^−1^ of Gb4 (Sigma, St Louis, MO, USA) for 72 h. To examine the involvement of the mitogen-activated protein kinase (MAPK) cascade or the transforming growth factor (TGF) β1 pathway in the induction of Epfn or Runx2 gene expression by Gb4, 50 μmol·L^−1^ of PD98059 (Cell Signaling Technology, Beverly, MA, USA) and 10 μmol·L^−1^ of SB203580 (Wako, Osaka, Japan) were added, respectively, to HAT cell culture media 1 h before Gb4 stimulation, followed by real-time quantitative polymerase chain reaction (RT-qPCR) analysis. Dimethyl sulphoxide (Sigma, St Louis, MO, USA) was added as a control.

### Tooth germ organ culture

An embryonic day 17.5 (E17.5) lower first molar from a mouse embryo was dissected under a stereomicroscope. The tooth germ was cultured on a floating Nucleopore membrane with or without Gb4 (0.5 μmol·L^−1^) in BGJb medium supplemented with Insulin-Transferrin-Sodium Selenite Supplement (ITS, Roche, Basel, Switzerland) and vitamin C (50 μg·mL^−1^; Supplementary Figure 1). After cultivation for 5 days, we investigated the expression of markers of ameloblast differentiation, as described previously.^15^

### Preparation of anti-Gb4 monoclonal antibody and immunohistochemistry

For immunohistochemistry, we prepared frozen (E16.5 and E18.5) and paraffin-embedded postnatal day 1 (P1) and P3 sections of mouse heads. A hybridoma clone cell line, BMR26, was obtained from RIKEN (Tsukuba, Japan)^30^ and cultured in RPMI-1640 medium (Invitrogen, Carlsbad, CA, USA) with interleukin (IL)-6 recombinant protein (0.7 ng·mL^−1^; Peprotech, Rocky Hill, NJ, USA). The hybridoma supernatants containing the anti-Gb4 antibody were concentrated and purified using a size fraction spin column. Sections were incubated with the anti-Gb4 primary antibody (purified from BMR26) and then visualized with an Alexa 488 conjugated anti-mouse IgG antibody (Invitrogen, Carlsbad, CA, USA). Nuclear staining was performed with 4′,6-diamidino-2-phenylindole dye (Invitrogen, Carlsbad, CA, USA).

### RNA isolation and RT-qPCR analysis

HAT-7 cells were cultured in 6-well plates at 2.0 × 10^5^ cells per well. Total RNA from the cells was purified with TRIzol reagent (Invitrogen, Carlsbad, CA, USA), and then 1 μg was reverse transcribed using the SuperScript VILO complementary DNA (cDNA) Synthesis kit (Invitrogen, Carlsbad, CA, USA). After cDNA synthesis, a real-time PCR analysis was performed using the StepOne kit with SYBR green PCR reagent (Applied Biosystems, Foster City, CA, USA). The qPCR mixtures, which contained 5 μL of a twofold dilution of the RT product and 0.2 μmol·L^−1^ of each primer, were incubated for 8 min at 95 °C and then subjected to 40 cycles of 95 °C for 30 s, 61 °C for 10 s, and 72 °C for 10 s. The sequences of the gene-specific primers are listed in Table 1.

### Western blotting

Cells were plated in 6-well plates at 3 × 10^5^ cells per well for 1 day before being assayed. After the cells were held in serum-free medium for 1 h, 1.0 μmol·L^−1^ of Gb4 was added to the medium. Thereafter, the cells were lysed with CytoBuster Protein Extraction Reagent (Novagen, Madison, WI, USA) containing Aprotinin (Roche, Basel, Switzerland), Leupeptin (Roche, Basel, Switzerland), and phenylmethenesulphonyl fluoride (Wako, Osaka, Japan) at 4 °C for 10 min. Lysates were clarified by centrifugation for 10 min at 13 000*g* and diluted with 4 × sample buffer, followed by incubation at 70 °C for 15 min. Specimens were separated using 4%–12% sodium dodecyl sulfate (SDS)–polyacrylamide gel electrophoresis (PAGE) and analysed by western blotting. After being blocked with skim milk, the blotted membrane was incubated with the primary antibodies overnight at 4 °C, and signals were detected by horseradish peroxidase-conjugated secondary antibodies (R&D Systems, Minneapolis, MN, USA) with an ECL Prime kit (Amersham Bioscience, Little Chalfont, UK). Western blotting was performed with anti-p44/p42 (extracellular signal-regulated kinase-1/2, ERK1/2), anti-phospho p44/p42 (p-ERK1/2; Tyr202/Tyr204), anti-p38 MAPK, and anti-phospho-p38 MAPK (Thr180/Tyr182) antibodies (Cell Signaling Technology, Beverly, MA, USA). A quantitative densitometric analysis of the western blotting results was conducted using ImageJ densitometry software (Version 1.6; National Institutes of Health, Bethesda, MD, USA).

### Cell proliferation assay

A 5-bromo-2′-deoxyuridine (BrdU) incorporation assay was performed using the BrdU labelling and detection kit (Roche, Tokyo, Japan) as described previously.^17^ The HAT-7 cells with BrdU incorporation were detected using an anti-BrdU mouse antibody and were visualized using fluoroscein isothiocyanate-conjugated anti-mouse IgG antibody (Roche, Basel, Switzerland). Cells were plated at 4 × 10^3^ cells per well in 96-well plates for 24 h, and then the cell numbers were determined by measuring the absorbance of the culture medium. For a cell proliferation assay, the cells were incubated at the same cell density described above for 24 to 72 h before the addition of Gb4 at concentrations ranging from 0 to 2 μmol·L^−1^. The number of viable cells was evaluated 1, 2, and 3 days after the initial treatment using a Cell Counting Kit-8 (WST-8; Dojindo, Kumamoto, Japan), according to the manufacturer's instructions. The optical absorbance at a wavelength of 450 nm was measured in the supernatant of each well using a plate reader (TECAN, Männedorf, Switzerland).

### Statistical analysis

Each independent experiment was repeated three times and representative findings are shown. The values obtained are shown as mean±standard deviation. The significance of differences between the control and treatment groups was evaluated by Student's *t*-test. *P*<0.05 were considered to indicate statistical significance.

## Results

### Localization of Gb4 in tooth development

We previously reported that there was GSL expression in dental epithelial cells and that Gb4 was strongly detected in the neutral fractions using thin-layer chromatography.^22^ To characterize which types of cells involved in tooth development express Gb4, we performed immunohistochemical analyses of developing incisors and molars using an anti-Gb4 (BMR26) antibody. Immunofluorescence staining did not reveal Gb4 expression in the tooth germs obtained on E16.5 or E18.5 (Figure 1b1 and 1b2). However, Gb4 was expressed in ameloblasts and odontoblasts in mouse molars on P3 (Figure 1b3 and 1b4), as well as in P1 and P3 mouse incisors (Figure 1b6). In addition, the enamel matrix protein Ambn was strongly expressed in differentiating dental epithelial cells (Figure 1b5). These results suggest that Gb4 is expressed in dental epithelial cells and is colocalized with Ambn, a marker of ameloblast differentiation. The expression of Gb4 in dental mesenchymal cells was interesting, and hence we investigated the function of Gb4 in odontoblasts.

### Gb4 has no effect on dental epithelial cell proliferation

Because Gb4 was found to be expressed by differentiating dental epithelial cells, we speculated that it may contribute to altering the cell state from proliferation to differentiation. To analyse the potential role(s) of Gb4 in dental epithelial cell proliferation, we examined HAT-7 cell proliferation with different concentrations of Gb4 added to the culture media. The results of BrdU incorporation and WST-8 assays revealed that dental epithelial cell proliferation was not affected by pretreatment with Gb4 (Supplementary Figure 2). These results suggest that Gb4 has no effect on dental epithelial cell proliferation.

### Gb4 induces ameloblast marker gene expression

**Figure 2:**
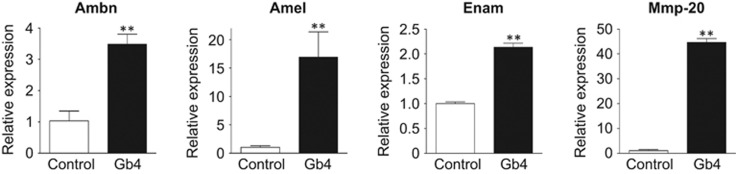
**Real-time qPCR analysis of ameloblast marker gene expression in HAT-7 cells.** The expression levels of Ambn, Amel, Enam, and Mmp-20 were examined. Glyceraldehyde-3-phosphate dehydrogenase (Gapdh) was used as an internal control. The expression levels of ameloblast marker genes in HAT-7 cells cultured with 0.5 μmol·L^−1^ of Gb4 for 72 h. Ambn, Amel, Enam, and Mmp-20 were enhanced in the presence of Gb4. **P<0.01. Ambn, ameloblastin; Amel, amelogenin; Enam, enamelin; Gb4, globoside; Mmp-20, matrix metalloproteinase-20; qPCR, quantitative polymerase chain reaction.

To investigate the biological functions of Gb4 expression in the dental epithelium, we examined the expression of ameloblast marker genes in HAT-7 cells treated with exogenous Gb4 in the culture media. A real-time PCR analysis revealed that Gb4 induced the expression of early marker genes of ameloblasts, such as Ambn (3.5-fold), Amel (16-fold), Enam (2-fold), and Mmp-20 (40-fold) (Figure 2). These results suggest that Gb4 promotes the differentiation of dental epithelial cells into ameloblasts.

**Figure 3:**
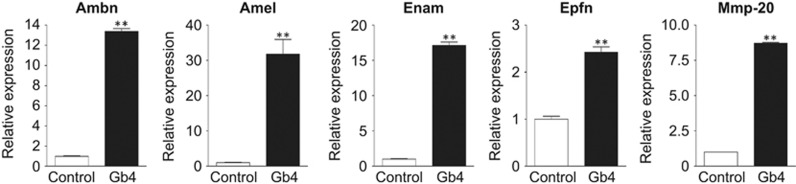
**Real-time PCR analysis of ameloblast marker gene expression in tooth germ.** The expression levels of ameloblast marker genes in tooth germs cultured with and without 0.5 μmol·L^−1^ Gb4 for 5 days. The expression of ameloblast marker genes was enhanced in the presence of Gb4. **P<0.01. Ambn, ameloblastin; Amel, amelogenin; Enam, enamelin; Gb4, globoside; Mmp-20, matrix metalloproteinase-20; qPCR, quantitative polymerase chain reaction.

An analysis of dental epithelial cells showed that Gb4 accelerates the differentiation of the dental epithelium. However, because the role of Gb4 in tooth development was unclear, we cultured tooth germs obtained on E17.5 with Gb4 for 5 days and analysed the dental cusp formation. We developed a mouse tooth germ organ culture system for this experiment. The real-time PCR findings revealed that Gb4 accelerated tooth germ cell differentiation, as shown by the remarkably promoted mRNA expression of the ameloblast markers Ambn, Amel, Enam, Epfn, and Mmp-20 (Figure 3). These results strongly suggest that Gb4 promotes the differentiation of inner enamel epithelial cells into ameloblasts.

### Gb4 accelerates NT-4-mediated dental epithelial cell differentiation

**Figure 4:**
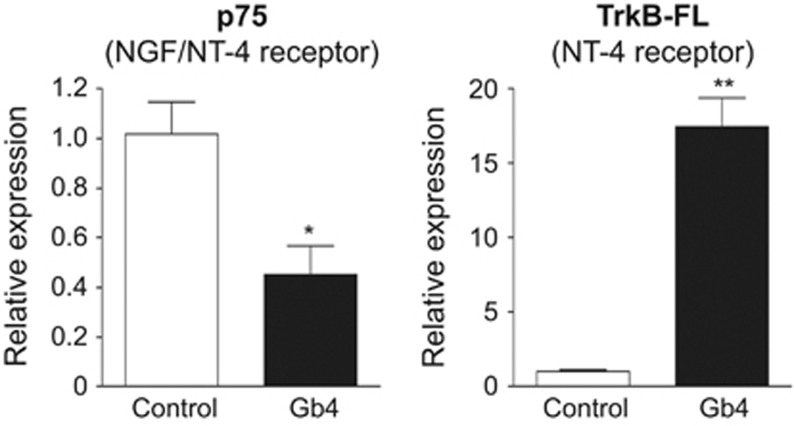
**The effects of Gb4 on the expression of the NT-4 receptors, p75 and TrkB-FL, in HAT-7 cells.** TrkB-FL induces NT-4-related Ambn expression, whereas the p75 receptor was expressed in undifferentiated dental epithelial cells. The results of a real-time PCR analysis of NT-4 receptor gene expression. In the presence of Gb4, the TrkB-FL expression was significantly upregulated, whereas that of p75 was downregulated. *P<0.05, **P<0.01. Gb4, globoside; NGF, nerve growth factor; NT, neurotrophin.

We previously reported that the presence of either GM3 or LacCer is essential for the activation of NT-4 signalling during the differentiation of dental epithelial cells, and promotes NT-4-mediated Ambn expression.^22^ In dental epithelial cells, NT-4 binds its receptor, TrkB, and upregulates Ambn expression by activating the ERK1/2 pathway.^21^ However, NT-4 also binds the p75 receptor that is expressed in undifferentiated dental epithelial cells. To further analyse the synergistic effects of Gb4 on NT-4-mediated dental epithelial cell differentiation, we examined the gene expression of the NT-4 receptor. A real-time PCR analysis revealed that the TrkB-FL expression was upregulated, whereas that of p75 was downregulated (Figure 4), suggesting that Gb4 stimulates NT-4 sensitivity and accelerates the differentiation of dental epithelial cells into ameloblasts.

### Gb4 activates the ERK and p38 MAPK cascades and enhances TGF-β1-mediated ERK1/2 phosphorylation in dental epithelial cells

**Figure 5:**
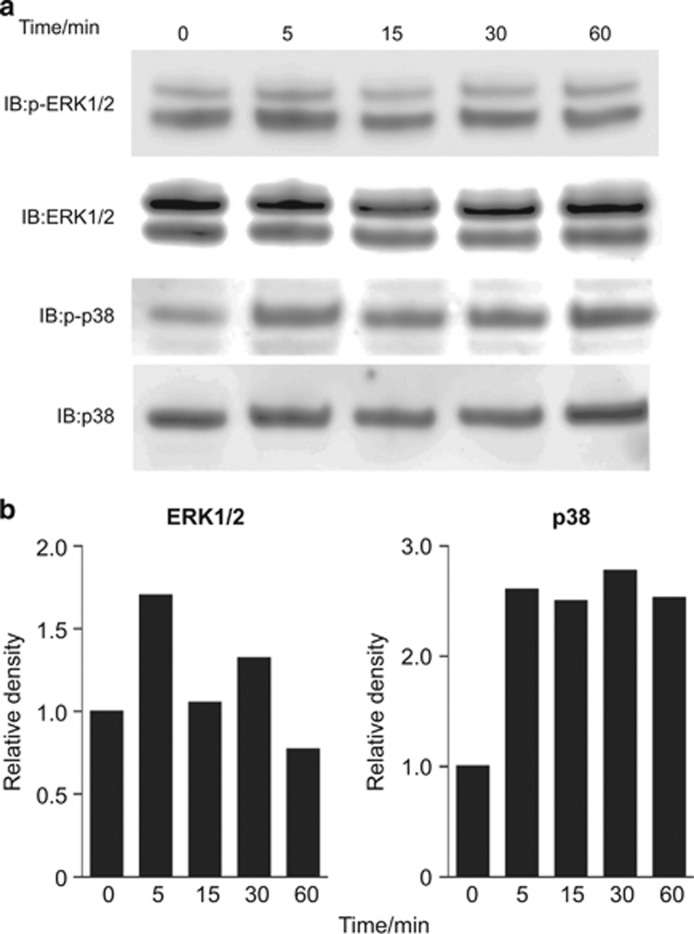
**The effects of globoside on the activation of signalling pathways.** Western blotting was performed to determine the effects of exogenous Gb4 on the phosphorylation of molecules in different signalling pathways. (**a**) HAT-7 cells were treated with 1.0 μmol·L^−1^ of Gb4 and were harvested after 0, 5, 15, 30, and 60 min. Lysates from cells treated with Gb4 were subjected to a western blot analysis with anti-ERK1/2 and anti-p38 antibodies. (**b**) Quantification was performed using densitometry with ImageJ software (National Institutes of Health, Bethesda, MD, USA). The relative density at 0 min was set at 1 for comparison. Gb4, globoside.

NT-4 stimulates dental epithelial cell differentiation into ameloblasts in part by activating the ERK1/2 pathway. To analyse the effects of Gb4 on the activation of signalling pathways that are involved in regulating Ambn gene expression, we performed a western blot analysis using anti-phospho ERK1/2 (p44/42) and anti-p38 MAPK antibodies. Upregulation of the phosphorylation of ERK1/2 was observed at 5 and 30 min after stimulation with Gb4 (Figure 5a and 5b). The phosphorylation of p38 was observed from 5 to 60 min. These results indicate that Gb4 constitutively activates the ERK and p38 MAPK cascades without any additional growth factors in HAT-7 cells.

### Gb4 induces Epfn and Runx2 *via* the ERK and p38 pathways

**Figure 6:**
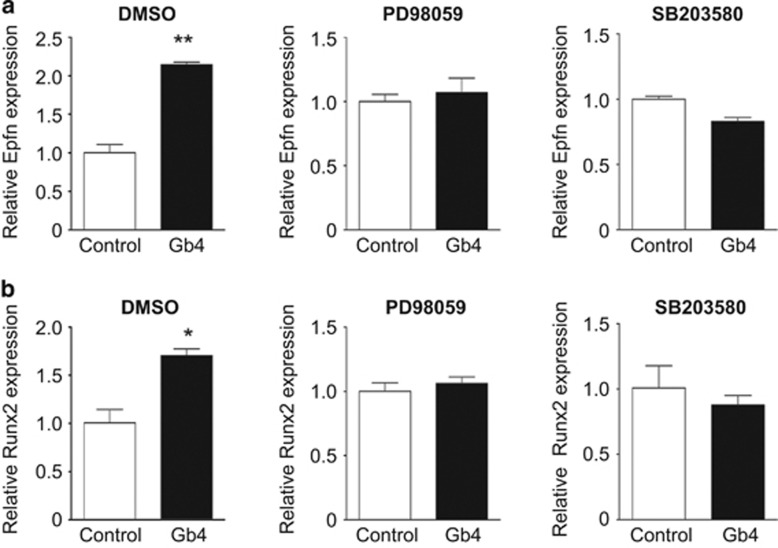
**Gb4 induces the expression of Epfn and Runx2 *via* the ERK1/2 and p38 MAPK pathways.** The results of a real-time qPCR analysis of (**a**) Epfn and (**b**) Runx2 in HAT-7 cells in the presence or absence of Gb4 with or without PD98059 (50 μmol·L^−1^, ERK signalling inhibitor) or SB203580 (10 μmol·L^−1^, p38 MAPK signalling inhibitor). *P<0.05, **P<0.01. Epfn, Epiprofin; ERK1/2, extracellular signal-regulated kinase-1/2; Gb4, globoside; MAPK, mitogen-activated protein kinase; qPCR, quantitative polymerase chain reaction.

Epfn and Runx2 have been identified as positive regulators for the transcription of the *Ambn* gene by binding to its proximal promoter. We found that exogenous Gb4 promoted Epfn expression in developing molars (Figure 3) as well as HAT-7 cells (Figure 6a). The induction of Epfn by Gb4 did not occur in the presence of either PD98059 or SB203580 that are ERK and p38 signalling inhibitors, respectively. The Gb4-mediated Ambn induction in HAT-7 cells was also abolished by treatment with PD98059 or SB203580 (data not shown). Taken together, these results suggest that Gb4 activates ERK and p38 signalling to enhance Epfn expression, and the induction of Epfn induces the differentiation of dental epithelial cells into ameloblasts that express Ambn. Similarly, ERK and p38 signal inhibitors blocked the upregulation of the gene expression *Runx2* by Gb4 (Figure 6b).

## Discussion

Our *in vitro* and *ex vivo* results showed that Gb4, a globo-series glycosphingolipid, promotes the differentiation of dental epithelial cells into ameloblasts, with no apparent effect on dental epithelial cell proliferation. The expression pattern of Gb4 during tooth development was limited to the dental epithelial cells that were differentiating into Ambn-expressing ameloblasts (Figure 1b), strongly suggesting that Gb4 is involved in this differentiation. Furthermore, analyses of the results that were obtained using the HAT-7 dental epithelial cell line, as well as studies of tooth bud organ culture, clearly showed the expression of numerous enamel matrix and enamel-specific protease genes was induced in the presence of Gb4 (Figures 2 and 3).

Several growth factors and extracellular matrices have been reported to promote ameloblast differentiation during tooth development.^31^ We previously found that a neurotrophic factor, NT-4, stimulates ameloblast differentiation and that NT-4-deficient mice showed a thin enamel matrix layer as a result of a downregulation of the expression of Ambn that encodes an enamel matrix protein and is a unique marker of ameloblasts.^21^ Thus, we investigated the molecular mechanism underlying Gb4 in NT-4 signalling during dental epithelial cell differentiation.

**Figure 7:**
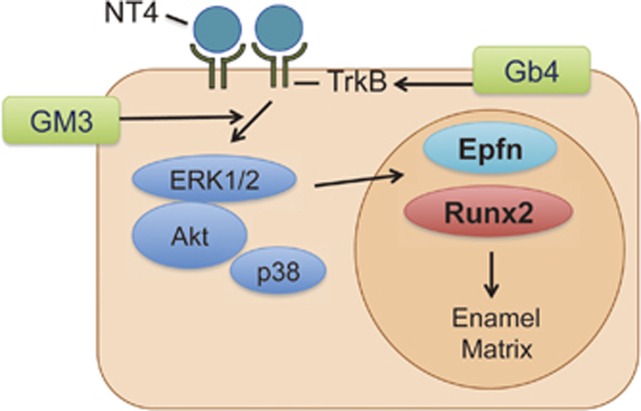
**The roles of Gb4 in dental epithelial cells.** GM3 stimulates the intracellular signalling for ameloblast differentiation, as described previously. On the other hand, Gb4 promotes the differentiation of dental epithelial cells into ameloblasts by upregulating TrkB-FL expression, leading to changes in the responses to various growth factors, such as NT-4. This leads to increased expression of epiprofin and Runx2 that are positive regulators of the ameloblastin gene. Gb4, globoside; NT, neurotrophin.

The NGF receptor, p75, is a marker of progenitor cells in ectodermal organs.^32, 33, 34^ During dental epithelial cell differentiation, its expression is elevated in immature proliferating dental epithelial cells and is decreased in pre-ameloblasts.^35^ Furthermore, the expression of p75 is undetectable in ameloblasts that do not divide.^35^ Dental epithelial cells express NGF as well as other neurotrophin factors, including NT-4.^36, 37, 38, 39^ The ligand of p75 in dental epithelial progenitor cells has not been well elucidated. However, it is clear that the function of NT-4 *via* TrkB is essential for normal ameloblast differentiation and the inhibition of dental epithelial cells.^21^ Thus, the conversion of neurotrophic factor receptors is a required step in normal ameloblast differentiation. Otherwise, NT-4 signalling via TrkB in dental epithelial cells is disrupted, and the dental epithelial cells will fail to properly differentiate into ameloblasts.^21^ Interestingly, we found that Gb4 blocked the expression of p75 in HAT-7 cells, whereas the expression of TrkB was induced by Gb4 in these cells. These results suggest that Gb4 induces the differentiation of dental epithelial cells into ameloblasts by changing their responsiveness to NT-4 (Figures 4 and 7).

In our previous study, a thin-layer chromatography analysis revealed dominant expression of Gb4 and GM3 in dental epithelial cells.^22^ GM3 stimulates dental epithelial cell differentiation into enamel matrix-secreting ameloblasts by enhancing the intracellular signalling of neurotrophic factor-4 *via* the TrkB receptor. We found that exogenous administration of GM3 reorganized the lipid raft structure and enhanced intracellular signal transduction, important for ameloblast differentiation.^22^

In a previous study, we revealed that in the dental epithelium, ERK phosphorylation was induced by NT-4, and this was necessary for Ambn expression.^21, 22^ In addition, p38 MAPK participates in tooth development, and the phosphatidylinositol-3-kinase signalling pathway plays an important role in the induction of the expression of Ambn.^40, 41^ In this study, we showed that Gb4 constitutively promoted the phosphorylation of ERK1/2 and p38 MAPK (Figure 5). These findings suggest that Gb4 promotes ameloblast differentiation through not only the NT4–TrkB–ERK signalling pathway but also the p38 MAPK signalling pathway. Furthermore, our results strongly suggest that Gb4 enhances the gene expression of *Epfn* and *Runx2*, both of which are important for the transcription of Ambn *via* activation of the ERK and/or p38 MAPK pathways. Taken together with our previous results, the present study shows the sequential and distinct roles of Gb4 and GM3 in dental epithelial cells undergoing differentiation into ameloblasts.

It has been reported that Runx2 induces the differentiation of preodontoblasts into immature odontoblasts at an early stage and that Runx2 regulates ERK/p38 MAPK phosphorylation for the commitment of mesenchymal cells osteoblasts.^42^ The expression of Gb4 was also detected in odontoblasts in postnatal incisors and molars (Figure 1b). Odontoblasts share characteristics with the osteoblast, including their gene expression profile, with both expressing proteins such as dentin sialophosphoprotein (Dspp), osteocalcin and Runx2.^43^ Our preliminary results showed that Gb4 promoted the differentiation of dental mesenchymal cells into odontoblasts by inducing the expression of Runx2 and Dspp (data not shown). Thus, Gb4 may regulate odontoblast differentiation through the activation of Runx2/ERK/MAPK signalling.

Our present findings provide evidence of a novel mechanism by which dental epithelial cells differentiate into ameloblasts, where Gb4 is involved in switching the expression of receptors for NT-4, a positive regulator of ameloblast differentiation. Gb4 expression triggers alterations in the dental epithelial cell state from proliferation to differentiation by enhancing their responsiveness to NT-4. This discovery opens doors to a more thorough investigation of the mechanism(s) that control GSL expression during tooth development.

## Figures and Tables

**Table 1:** The sequences of the gene-specific primers used for the real-time qPCR analysis

Gene	Forward primer	Reverse primer	Size/bp
Mouse/rat *Amel*	5′-GATGGCTGCACCACCAAATC-3′	5′-CTGAAGGGTGTGACTCGGG-3	65
Mouse *Enam*	5′-TGCAGAAATCCGACTTCTCCT-3′	5′-CATCTGGAATGGCATGGCA-3′	114
Rat *Enam*	5′-ACCGAAAGTCCTGACACATTGG-3′	5′-CCTGAAGCAGTAAACAGCCGTG -3′	154
Mouse/rat *Mmp-20*	5′-GGCGAGATGGTGGCAAGAG-3′	5′-CTGGGAAGAGGCGGTAGTT-3′	166
Mouse *Ambn*	5′-ATGAAGGGCCTGATCCTGTTC-3′	5′-GTCTCATTGTCTCAAGGCTCAAA-3′	130
Mouse/rat *Epfn*	5′-CCTTTCTATTTCACCCTCCCCTG-3′	5′-CCACCACCTCATCTCTGCTTTCTC-3′	282/248
Rat *Runx2*	5′-CGGGAATGATGAGAACTACTC-3′	5′-TGAAACTCTTGCCTCGTCC-3′	118
Mouse/rat *GAPDH*	5′-CCATCACCATCTTCCAGGAG -3′	5′-GCATGGACTGTGGTCATGAG-3′	322

Ambn, ameloblastin; Amel, amelogenin; Enam, enamelin; Epfn, epiprofin; GAPDH, glyceraldehyde-3-phosphate dehydrogenase; Mmp, matrix metalloproteinase; qPCR, quantitative polymerase chain reaction; Runx2, Runt-related transcription factor 2.

The real-time qPCR analysis was performed using a StepOne kit with SYBR green PCR reagent with the listed primers.
