# Supplementary figures and images for: Globoside accelerates the differentiation of dental epithelial cells into ameloblasts
Source: Int J Oral Sci. 2016 Oct 21;8(4):205–12. doi: 10.1038/ijos.2016.35 (PMC5168416; doi:10.1038/ijos.2016.35)

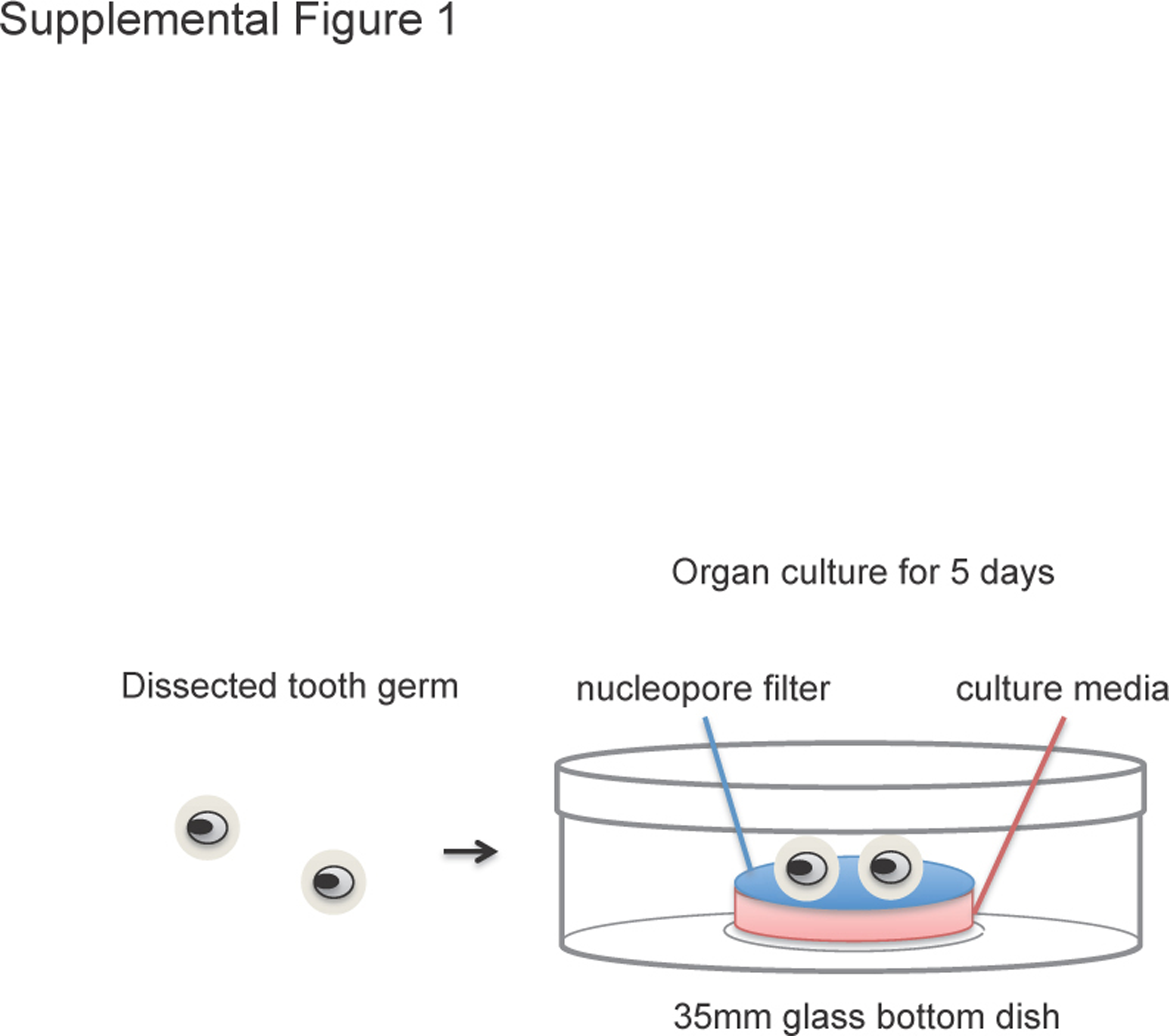

Supplement: Supplementary Figure 1 [file ijos201635x1.tif]

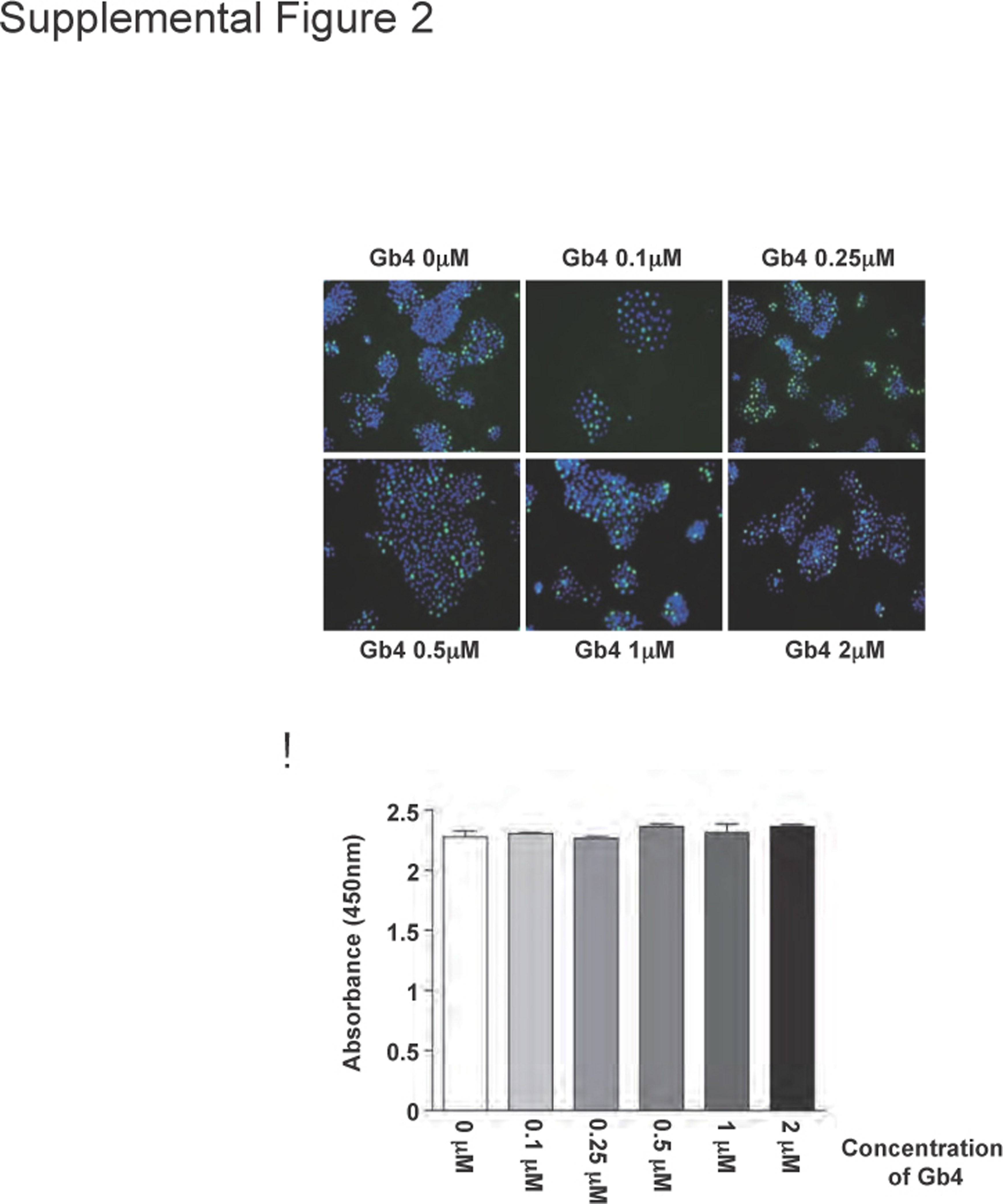

Supplement: Supplementary Figure 2 [file ijos201635x2.tif]
