# Supplementary material for: Globoside accelerates the differentiation of dental epithelial cells into ameloblasts
Source: Int J Oral Sci. 2016 Oct 21;8(4):205–12. doi: 10.1038/ijos.2016.35 (PMC5168416; doi:10.1038/ijos.2016.35)
Supplement: Supplementary Figure Legends [file ijos201635x3.docx]

**Supplemental Figure 1. The organ culture system used in the study.**

**Supplemental Figure 2. The effects of Gb4 on the proliferation of dental epithelial cells.**

Dental epithelial cells (HAT-7) were cultured with various concentrations of Gb4. (A) Fluorescence microscopic images of cells left to incorporate BrdU for one hour with and without Gb4 stimulation. (B) HAT-7 cells were cultured with various concentrations of Gb4. The cellular proliferation was determined using a WST-8 kit. The columns show the ratio of cells at an OD of 450 nm.
